# Strong laser field control of fragment spatial distributions from a photodissociation reaction

**DOI:** 10.1038/s41467-017-01139-6

**Published:** 2017-11-07

**Authors:** María E. Corrales, Rebeca de Nalda, Luis Bañares

**Affiliations:** 1 Departamento de Química Física (Unidad Asociada de I+D+i al CSIC), Facultad de Ciencias Químicas, Universidad Complutense de Madrid, 28040 Madrid, Spain; 2 Instituto de Química Física Rocasolano, CSIC, C/ Serrano 119, 28006 Madrid, Spain

## Abstract

The notion that strong laser light can intervene and modify the dynamical processes of matter has been demonstrated and exploited both in gas and condensed phases. The central objective of laser control schemes has been the modification of branching ratios in chemical processes, under the philosophy that conveniently tailored light can steer the dynamics of a chemical mechanism towards desired targets. Less explored is the role that strong laser control can play on chemical stereodynamics, i.e. the angular distribution of the products of a chemical reaction in space. This work demonstrates for the case of methyl iodide that when a molecular bond breaking process takes place in the presence of an intense infrared laser field, its stereodynamics is profoundly affected, and that the intensity of this laser field can be used as an external knob to control it.

## Abstract (web summary)

Strong laser light can intervene and modify the dynamical processes of matter. Here, the authors show how an intense laser field affects the spatial distribution of fragments in a molecular bond-breaking process, and how the intensity of this laser field can be used as an external knob to control it.

## Introduction

Stereodynamics, understood as the study of the directional behaviour of matter, is essential to describe photon and particle collision phenomena. If we take molecular photodissociation as an example, the microscopic level description of the process fundamentally includes the knowledge of vectors like the polarization of the light field, the transition dipole moment of the molecule and its angular momentum, the velocity vectors of the fragments and their final angular momenta. Angular momentum theory is the framework for the description of those vector quantities and the correlations between them^1, 2^.

The angular distribution of fragments resulting from a molecular bond breaking process induced by linearly polarized light reveals the preferential directions of fragment ejection in the laboratory frame. These are only a faithful reflection of the nature of the transition that causes dissociation in the axial recoil limit and in the absence of fragment alignment. Thus, the three mentioned ingredients (preferential molecular excitation due to the nature of the transition, loss of anisotropy due to rotation of the molecular axis during dissociation, and fragment alignment) contribute to the observed reaction stereodynamics.

Even though these complex molecular processes are light-induced, the description of their dynamics is essentially field-free, in the sense that they are governed by the molecular Hamiltonian. However, when a light field of high intensity is added, the matter states become “dressed by light” and we move into the field of laser control, or, from an alternative view, the dynamics in the presence of strong laser fields, a theme that has experienced tremendous advances over recent years. When a reaction takes place immersed in light intense enough to reshape the potential energy surfaces, both electrons and nuclei in the system can evolve in new directions. The essential tool in these schemes is a control laser pulse that is precisely defined in terms of spectrum, time, intensity and polarization. A significant number of theoretical proposals and experimental demonstrations of laser control have been presented^3–6^ showing laser-induced modifications of observables like the absorption spectrum^7–10^, lifetimes^9, 11–13^, quantum yields^14–16^ or even spatial localization of molecules in space^17^.

Precedents on the subject of the control of the angular character of a molecular process using light are not very abundant. An important exception concerns the theme of gas-phase molecular alignment through the use of polarized laser fields, which has received very intense attention mainly from the beginning of the 21st century^18–21^. Indeed, photofragment angular distributions have been controlled through this mechanism^22^. With a related approach, fragment asymmetries of the CO product could be forced by hexapole orientation of the parent OCS molecule^23^. In relation with this, an interesting proposal has been offered^24^ where the effect of a laser-induced conical intersection on the time-dependent alignment of diatomic molecules has been explored. On a different approach, significant work has been devoted to create asymmetries in the fragment spatial distributions by the introduction of asymmetric laser fields, either few-cycle carrier envelope phase (CEP)-stabilized fields^25^, or bichromatic fields with relative phase control^26, 27^.

**Fig. 1:**
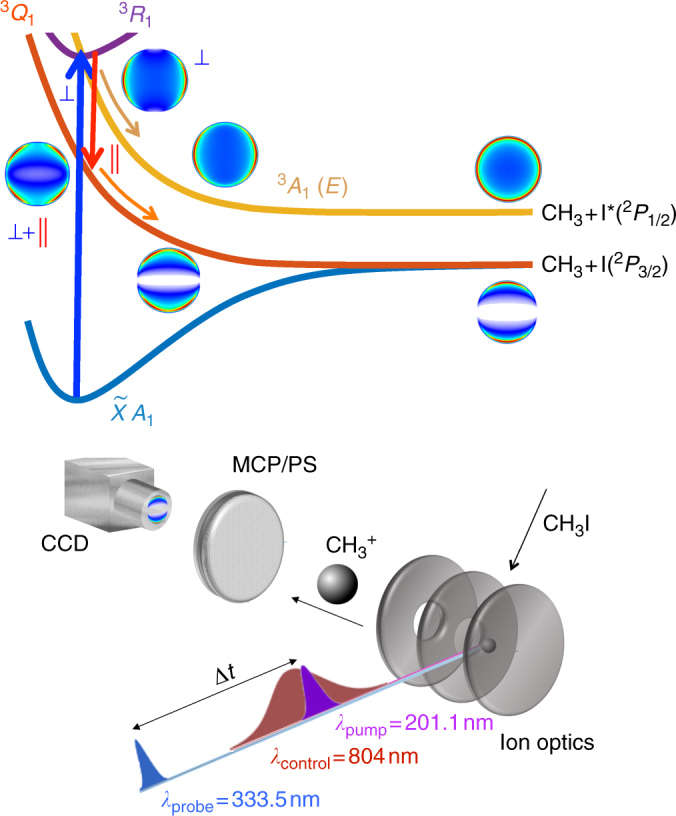
Laser control scheme of fragment spatial distributions of a predissociation reaction. (Top) Cuts of the potential energy surfaces involved along the C-I coordinate, and graphical indication of the dynamics. After excitation to the ^3^ *R*_1_ Rydberg state, the field-free predissociation channel occurs through crossing with the dissociative ^3^ *A*_1_(*E*) state (orange route). The presence of a low intensity NIR field opens a pump-dump channel by coupling to the ^3^ *Q*_1_ state (red route). Examples of the time-resolved angular distributions of the methyl fragments born through these two channels are indicated in the Figure. (Bottom) Sketch of the experiment (see Methods), with the three laser pulses employed (pump, control, probe), the interaction region with the molecular beam and the electrode setup designed for extraction and velocity map detection

In previous work performed in our group, we treated the case of an electronic predissociation process in the methyl iodide (CH_3_I) molecule. CH_3_I possesses a strong absorption band, in the spectral region of 200 nm, known as the *B*-band, which corresponds to transitions from a nonbonding 5*pπ* electron of the iodine atom to a 6*s* molecular Rydberg orbital (see Fig. 1). This state is bound, but the molecule predissociates due to crossings with repulsive states. In Fig. 1, this route corresponds to vertical excitation to the ^3^ *R*_1_ Rydberg state, depicted in dark violet, followed by dissociation along the ^3^ *A*_1_ surface, depicted in orange. The absorption spectrum, as a result, shows clear vibrational structure with peaks that are lifetime broadened^28–32^. Alekseyev et al.^33^ recently published ab initio potential energy curves and transition moments on this system in one dimension. The work found that the *B*-band is dominated by the perpendicular ^3^ *R*_1_ ← $\tilde X$ *A*_1_ transition and that the main predissociation channel occurs via interaction with the repulsive 4*E*(^3^ *A*_1_) state, which asymptotically yields spin-orbit excited iodine atoms: I*(^2^ *P*_1/2_) + CH_3_. Recent experimental work has explored this process^34–37^ focusing on the real-time details of this relatively fast predissociation. In particular, a lifetime of 1.5 ± 0.1 ps was measured for the ground vibrationless level of the ^3^ *R*_1_ Rydberg state^35^. Additional findings of that work were a quantum yield of unity for the generation of spin-orbit excited I*(^2^ *P*_1/2_), and iodine images registered at short times (<500 fs) after excitation, which revealed strong anisotropy, confirming the perpendicular character of the transition.

As was mentioned above, three ingredients must be taken into account for the description of the angular distribution of photofragments resulting from molecular bond fission: selective excitation, parent molecule rotation and fragment alignment. The case of the *B*-band of CH_3_I clearly shows the role of this combination of elements. The I*(^2^ *P*_1/2_) fragments cannot show alignment, and their angular distribution, measured at very short times after molecular excitation^35^, accurately reflected the preferential excitation of molecules whose molecular axis was perpendicular to the light polarization axis (anisotropy parameter, *β* ≈ −1). For the CH_3_ counterpart, a thorough theoretical and experimental study^38^ revealed the role of molecular excited-state lifetimes, parent and fragment rotation and fragment angular momentum alignment on the time-dependent stereodynamics observed.

Here, we demonstrate that the angular distribution of fragments originating from a photodissociation process is controllable through the addition of an intense near-infrared (NIR) laser, in a relatively simple scheme (see Fig. 1) where the control knob through which the fragment spatial distribution is modified is simply the intensity of this NIR laser. Thus, we base our procedure on a standard short-pulse pump-probe scheme where the pump laser excites an allowed molecular transition and the probe laser later ionizes the resulting fragments, with velocity map imaging detection. To this scheme we add a moderately intense laser, in the NIR region of the spectrum, which temporally overlaps with the excitation event, and we show that the presence of this laser field causes important changes in the fragment distribution in space, and that its intensity can tailor the details of such distribution. The case study is the methyl iodide molecule, for which it is shown that upon carbon-iodine bond fission the distribution of methyl fragments in space can be tailored through a moderately intense picosecond near-infrared laser field.

## Results

### Field-free conditions

**Fig. 2:**
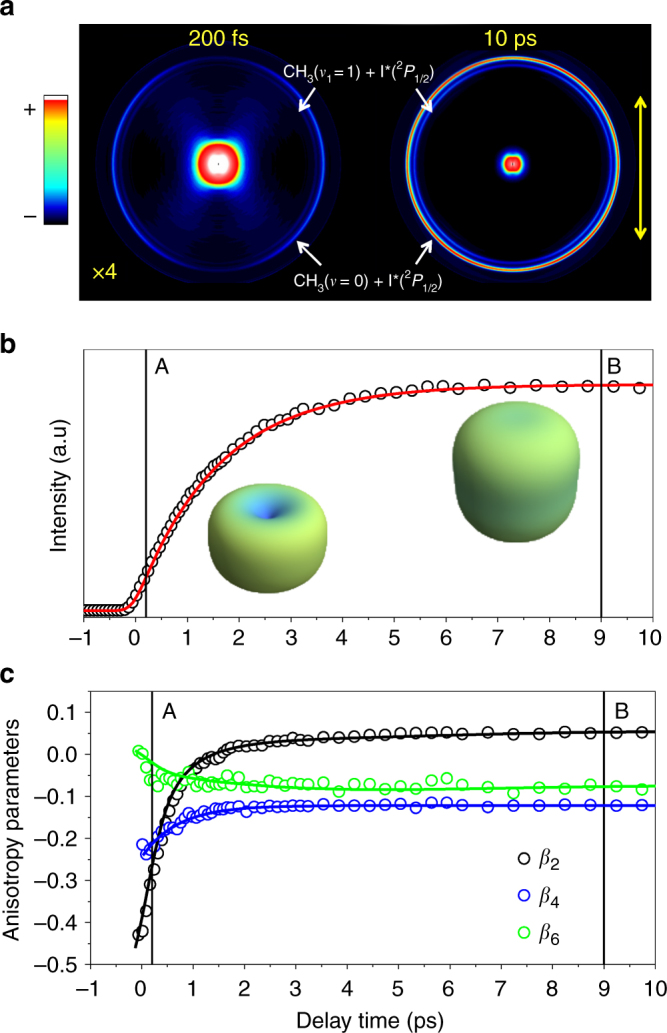
Angular distributions of methyl fragments in the absence of external fields. **a** Abel-inverted velocity map images of the methyl fragment resulting from CH_3_I *B*-band predissociation acquired 200 fs (left) and 10 ps (right) after excitation; the central structure is due to multiphoton ionization processes. Two well-defined rings appear in the images. The outer ring corresponds to vibrationless CH_3_(*ν* = 0) formed in correlation with spin-orbit excited I*(^2^ *P*_1/2_). The inner ring corresponds to the channel yielding symmetric stretch mode *ν*_1_ excited CH_3_ (*ν*_1_ = 1) in correlation with the I*(^2^ *P*_1/2_) fragments. The double arrow shows the direction of the linear polarization of the two laser pulses. **b** Total intensity transient of the vibrationless methyl fragment signal as a function of pump-probe time delay. 3D plot insets are polar representations of the angular distributions at point A (200 fs) and B (10 ps) of the delay scan; **c** Anisotropy parameters *β*_2_, *β*_4_ and *β*_6_ measured as a function of pump-probe time delay

Figure 2 shows the main features observed for the CH_3_ fragment arising from the predissociation of CH_3_I excited at the origin of the *B*-band at 201.1 nm in a field-free study. Figure 2a shows two Abel-inverted images of CH_3_ fragments resulting from the process, one acquired at early pump-probe time delays (200 fs) and the other well after predissociation is complete (10 ps, “asymptotic” temporal delay). The rings observed correspond to the channels yielding vibrationless CH_3_(*ν* = 0) (outer, intense) and symmetric stretch mode excited CH_3_(*ν*_1_ = 1) (inner, weak) both in correlation with spin-orbit excited I*(^2^ *P*_1/2_), due to predissociation through the ^3^ *A*_1_(4*E*) state^35^. No evidence is found for production of CH_3_ in correlation with ground spin-orbit state I(^2^ *P*_3/2_), which should appear as a larger velocity ring^35^. Figure 2b shows the global CH_3_(*ν* = 0) fragment signal, obtained by angular integration of the outer ring in the CH_3_ ion images, as a function of time delay, which reveals the exponential growth derived from the 1.5 ± 0.1 ps lifetime^35^ of the initially populated Rydberg state. 3D plot insets correspond to a polar representation of the angular distribution at positions A (200 fs) and B (10 ps) of the time delay scan. These measurements are associated to a one-photon excitation of CH_3_I and a (2 + 1) resonance enhanced multiphoton ionization (REMPI) detection of CH_3_. In this situation the CH_3_ fragment angular distribution can be expressed by an expansion over Legendre polynomials *P*_*i*_(cos *θ*) with the form

1 $$I\left( \theta \right) = \frac{\sigma }{{4\pi }}\mathop {\sum}\limits_{i = 0}^{2n + 2} {{\beta _i}{P_i}\left( {\cos \,\theta } \right)}$$

where *θ* is the angle between the recoil direction and the pump laser polarization, *σ* is the absorption cross section and *β*_*i*_ are anisotropy parameters^2^. In the present case (linearly polarized probe laser^39^) the *i* index can only take even values and *n* = 2 for a (2+1) REMPI, thus the distribution is completely described by three parameters: *β*_2,_ *β*_4_ and *β*_6_. Figure 2c contains these anisotropy parameters as a function of the time delay with respect to the excitation event.

It is clear that during the course of the process the observed angular character of the CH_3_(*ν* = 0) fragment distribution suffers important changes: the strong perpendicular character observed at short time delays is quickly lost. A detailed description of the reasons for this behaviour was recently presented^38^, where it was found to be the result of parent molecule and methyl fragment rotations, as well as methyl fragment angular momentum alignment.

### Strong field control process

On a different direction, a laser control scheme on this predissociation process was demonstrated^9^. Dynamic Stark control (DSC)^14^ was shown to cause resonance shifts in the *B*-band of CH_3_I^10^, and the combination of this effect with pump-dump strategies^40, 41^ was capable of causing dramatic changes in the predissociation lifetime and the product branching ratio. Indeed, the introduction of an intense infrared field opened new dynamical channels for the initially populated Rydberg state of the molecule, the most interesting of which was a pump-dump channel coupling the initially populated ^3^ *R*_1_ Rydberg state with the ^3^ *Q*_1_ dissociative surface (see Fig. 1), which yields vibrationless CH_3_(*ν* = 0) and ground spin-orbit I(^2^ *P*_3/2_). No evidence for a pump-dump process yielding CH_3_ in correlation with I*(^2^ *P*_1/2_) was observed, which reveals that the dominant pump process is mediated only by the coupling between the ^3^ *R*_1_ and the ^3^ *Q*_1_ dissociative surfaces by the intense NIR pulse^9^. A notable finding of that work was the possibility to control the lifetime of the predissociative state, which could be continuously “tuned” by controlling the intensity of the control field in the TW/cm^2^ range.

**Fig. 3:**
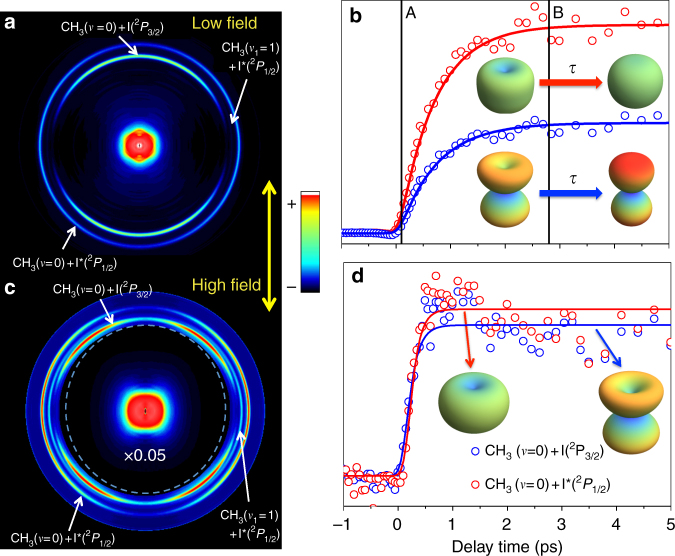
Angular distributions of methyl fragments under the influence of external NIR fields. **a** Abel-inverted image of the methyl fragment resulting from CH_3_I *B*-band predissociation at asymptotic pump-probe delay and for NIR field intensity of 0.5 TW/cm^2^. **b** Global intensity pump-probe temporal behaviour of the signal corresponding to the two channels yielding CH_3_(*ν* = 0) (direct and pump-dump, see text for details) observed in **a**. 3D insets are polar plots of the angular distributions of each channel at delays A and B. **c** Abel-inverted image of the methyl fragment resulting from CH_3_I *B*-band predissociation at asymptotic pump-probe delay and for NIR field intensity of 2.5 TW/cm^2^. The intensity of the central part has been multiplied by 0.05 for better visual inspection of the channels of interest. **d** Global intensity pump-probe temporal behaviour of the signal corresponding to the two channels yielding CH_3_(*ν* = 0) (direct and pump-dump, see text for details) observed in **c**. 3D insets are polar plots of the angular distributions of each channel at the delay indicated on the Figure. The images in **a**, **c** show three rings. The outer and middle rings correspond to vibrationless CH_3_(*ν* = 0) and symmetric stretch mode excited CH_3_(*ν*_1_ = 1), respectively, both in correlation with I*(^2^ *P*_1/2_) fragments. The inner ring corresponds to the pump-dump channel yielding CH_3_(*ν* = 0) and ground spin-orbit state I(^2^ *P*_3/2_). The double arrow shows the direction of the linear polarization of the three laser pulses

Here we explore the role played by an intense NIR laser field on the angular character of the photodissociation process as observed through the angular distributions of the CH_3_ fragments. For the experiments described here the pump laser pulse is fixed at −1 ps time delay with respect to the maximum intensity of the control laser pulse, maintaining the polarization axes parallel. Figure 3 contains the results obtained in two extreme cases with respect to the control laser intensity, the top row corresponding to what we shall call “low fields” (0.5 TW/cm^2^) and the bottom row, to “high fields” (2.5 TW/cm^2^). In the top row, the image on the left is the Abel-inverted CH_3_ fragment image obtained when a moderate-intensity (0.5 TW/cm^2^), ps-duration NIR pulse is present at the time of UV excitation. Resonant probing of the resulting CH_3_ fragments is done asymptotically. With respect to the image on the right of Fig. 2a, the main change is the appearance of the new dissociation pump-dump channel yielding CH_3_(*ν* = 0) + I(^2^ *P*_3/2_) resulting from the NIR field coupling the initially populated ^3^ *R*_1_ Rydberg state with the ^3^ *Q*_1_ dissociative surface. The reflection of this channel on the CH_3_ image is a lower kinetic energy ring, which shows strong parallel character, a consequence of the nature of the ^3^ *R*_1_ - ^3^ *Q*_1_ dump transition. Figure 3b shows the temporal evolution of the intensity of the predissociation and pump-dump channels, with a common risetime of 0.75 ps (hardly reduced with respect to the 1.5 ± 0.1 ps measured in the field-free case). 3D plot insets are polar representations of the angular distributions through each channel (red, predissociation CH_3_(*ν* = 0) + I*(^2^ *P*_1/2_), and blue, pump-dump CH_3_(*ν* = 0) + I(^2^ *P*_3/2_)) at pump-probe time delay positions A and B. Supplementary Fig. 1 shows the corresponding *β*_2,_ *β*_4_ and *β*_6_ anisotropy parameters for the two channels (predissociation and pump-dump) as a function of pump-probe time delay under the lower intensity NIR field. As was the case in the field-free situation, the angular distribution in the main CH_3_(*ν* = 0) + I*(^2^ *P*_1/2_) predissociation channel becomes more isotropic with time. The pump-dump CH_3_(*ν* = 0) + I(^2^ *P*_3/2_) channel, initially depleted at the poles due to the angular selection in the excitation process, develops a purely parallel character for longer time delays.

For the results shown in the bottom row of Fig. 3, a higher intensity (2.5 TW/cm^2^) ps-duration NIR field is applied synchronously with excitation. The image in Fig. 3c is the corresponding asymptotic Abel-inverted methyl fragment image. The excitation laser wavelength had to be retuned in this case to 200.6 nm (see Experimental section) to recover the resonance due to the Stark shift induced by the high intensity field^9, 10^. The same two channels described for the previous case in the above paragraph (predissociation CH_3_(*ν* = 0) + I*(^2^ *P*_1/2_), and pump-dump CH_3_(*ν* = 0) + I(^2^ *P*_3/2_)) are present now, but their angular character is notably different, the predissociation channel presenting a much more pronounced perpendicular character at asymptotic times and the pump-dump channel showing a lobular character with maxima at ±45° with respect to the laser polarization direction. Figure 3d shows the integrated value of the two channels as a function of the pump-probe delay, with a much-reduced lifetime (0.15 ps). The perpendicular and lobular characters of the predissociation and pump-dump channels, respectively, are clearly visible in the 3D polar insets. The corresponding *β*_2,_ *β*_4_ and *β*_6_ anisotropy parameters for the two channels (predissociation and pump-dump) as a function of pump-probe time delay under the higher intensity NIR field are depicted in Supplementary Fig. 2.

**Fig. 4:**
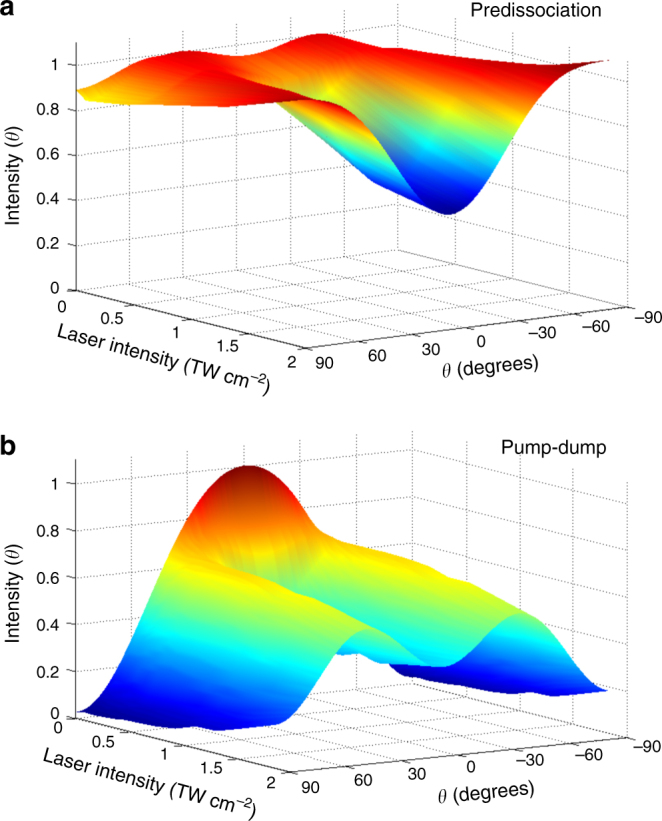
Dependence of fragment spatial distributions with control laser intensity. Asymptotic angular distributions of the methyl fragment resulting from *B*-band predissociation of CH_3_I as a function of the intensity of a NIR control field of picosecond duration present during UV excitation. **a** The top panel corresponds to the main predissociation channel and **b** the bottom panel to the pump-dump channel

The complete asymptotic angular distributions of the methyl fragment arising from *B*-band dissociation of CH_3_I are shown in Fig. 4, plotted as a function of the intensity of the ps NIR laser field used in temporal overlap with the excitation pulse. For the predissociation CH_3_(*ν* = 0) + I*(^2^ *P*_1/2_) channel (Figure 4a), characterized by a field-free quasi-isotropic distribution, higher NIR intensities cause an increasingly perpendicular character, with detected fragments concentrated around the equator of the Newton sphere. The angular distribution of fragments produced through the pump-dump CH_3_(*ν* = 0) + I(^2^ *P*_3/2_) channel (Figure 4b) suffers more dramatic changes with the NIR intensity, evolving quickly from a purely parallel distribution at near-zero NIR intensity to a pronounced lobular structure with the global minimum still at −90° and + 90° but a significant local minimum at 0°.

## Discussion

From the above results, it is clear that the intensity of the NIR field can be regarded as a control knob of the final detected angular distributions of the CH_3_ fragments resulting from the dissociation process studied in this work. It is our understanding that the mechanism responsible for this control is linked essentially to the aperture of new dynamical channels after Rydberg state excitation, especially the pump-dump channel described above. This causes two simultaneous effects that lie at the root of the angular control effect: the first is a reduction of the lifetime of the excited state; the second, a further angular selection related to the character of the dump process that not only determines the angular distribution for this channel but also selectively depletes population from the predissociation channel, thus producing significant angular changes in it.

The first (temporal) effect, in its extreme form (i.e. high intensity, causing a decrease of the excited state lifetime from its field-free value of 1.5 ps to values of the order of 150 fs) strongly reduces the loss of angular information occurring through rotation during the lifetime of the excited state, and this allows to recover the equatorial preference in the fragment distribution that mimics the original angular selection in a perpendicular transition. This effect can explain the main features of the distribution observed for the main channel at high NIR intensities. The NIR field polarization has a direct effect on the angular distribution observed for the pump-dump channel, due to the parallel character of the dump transition. For the case of low intensities (0.5 TW/cm^2^), where the predissociation channel is essentially isotropic, this parallel character is directly reflected on the angular distribution of fragments. However, the short-lived, perpendicularly selected population in the excited state resulting from a high intensity NIR field (2.5 TW/cm^2^) causes a lobular structure in the angular fragment distribution, peaking around 45°.

At this point, it is worth discussing the limits of the approach described in this work. For the conditions of the present experiments, taking the field-free distributions as the starting point, and considering that lifetime reduction and angular selection are the only factors affecting the distributions, the flexibility in the dump channel would be restricted between the limiting case of a purely parallel distribution for low control intensity to a significantly lobular distribution at high intensity. For the predissociation channel it will depend on the probability of the multiphoton ionization and dump channels, which in turn depends on the intensity of the control laser. For instance, we can modify the angular distribution from practically isotropic to strongly perpendicular. However, we believe that in practical terms, higher NIR field intensities than those used in this work would not achieve a significantly higher degree of control in this particular process, in relation to several factors: one is the increasing importance of multiphoton ionization channels that compete more favourably with the desired routes for higher intensities; the second is the significant Stark shift observed^9, 10^ for the initial excitation resonance at intensities higher than 0.7 TW/cm^2^. Thus, above this intensity it would be required to retune the excitation wavelength; the third is related to the fact that at the highest intensity used in this work, the reduction of the lifetime of the excited state is so significant that no further angular changes due to this mechanism are expected. Whereas we believe that the role of intensity has been in essence completely explored, polarization may be used as an additional knob for control. The results above were achieved with a linearly polarized NIR field oscillating along the same axis of the polarization of the excitation field. Due to the angular selectivity of the dump process, it is expected that more sophisticated, even to some extent tailored angular distributions might be achieved through the use of different polarization states for the NIR field. For instance, it would be straightforward to break the cylindrical symmetry of the angular distributions of the fragment simply by rotating the angle of the control polarization with respect to the pump polarization, thus creating more complex 3D angular distributions.

It is important to clarify to what extent the mechanism proposed in this paper is applicable to other systems or processes. We believe that one of the main results, the reversal of the loss of angular information due to rotation of the molecular axis during the lifetime of an excited state, is directly applicable to most analogous situations. The main requirement is that an intensity window is found where the probability of new post-excitation channels induced by the control field is high enough to cause significant reduction in the excited state lifetime without completely depleting the main dynamical channel or causing extensive ionization. We believe that this approach will be most favourable for lifetimes of the order of picoseconds; shorter lifetimes (femtosecond time domain) do not typically cause a significant loss of information due to molecular rotation for medium-sized systems, whereas for longer lifetimes (nanosecond region), extensive depletion of the main channels would be required to significantly reduce the effect of axis rotation. The second part of the mechanism, related to the pump-dump process, is in the present case linked to a specific allowed transition with a particular angular selectivity, which enriches the control scenario. Therefore, the search for an analogous response in a different system would require finding the optimum spectral region for the control pulse.

This laser control scheme of spatial distributions of fragments in photodissociation can find applications to reactions where the initial geometry has constraints. Two classes of reactions appear most promising: reactions in clusters and bimolecular reactions between species adsorbed on surfaces. In clusters, the range of trajectories and impact parameters is greatly limited by the equilibrium geometries, and the control of the fragment angular distributions upon dissociation of a member of the cluster may allow control of the reaction outcome. A similar argument applies to chemical reactions on surfaces, where typically dissociation fragments suffer encounters with co-adsorbate molecules, giving rise to new reaction products. The control of these processes may allow an increased degree of control on surface chemistry, including catalytic processes.

This work has demonstrated significant control on the stereodynamics of a predissociation process in CH_3_I through the introduction of a moderately intense, linearly polarized near-infrared laser field of picosecond duration. The main mechanism for control is the laser-induced reduction of the molecular excited state lifetime, which eliminates the loss of angular information of the initially selected molecular orientations upon UV excitation. Additionally, the angular selection characteristic of the new pump-dump channel induced by the NIR field enriches the possibilities for angular control in this case. The situation should be straightforward to generalize to other molecular processes where the dynamics are governed by the (picosecond) lifetime of an excited state.

## Methods

### Laser layout

The experiment requires the use of three laser pulses in a pump-control-probe scheme. All three pulses are generated from a chirped-pulse amplified Ti:sapphire laser producing 3.5 mJ pulses of 50 fs duration at 1 kHz repetition rate with a central wavelength of 804 nm. This fundamental pulse is split into three arms for control and manipulation. The first pulse is generated through frequency quadrupling, thus obtaining a central wavelength of 201.2 nm, a full width at half maximum (FWHM) bandwidth of ≈−0.3 nm and typical pulse energy of <1 μJ. This is the pump pulse causing excitation in the methyl iodide (CH_3_I) molecule from the vibrationless ground state to the ^3^ *R*_1_ Rydberg state in its vibrationless level. The probe pulse is produced through frequency quadrupling the output of an optical parametric amplifier (OPA). It has a central wavelength of 333.5 nm, a FWHM bandwidth of ≈−1.7 nm and typical pulse energy of 3–6 μJ, and it is employed to ionize CH_3_ fragments via a 2 + 1 resonance enhanced multiphoton ionization (REMPI) scheme. Finally, in the third (“control”) arm, the fundamental near-infrared pulse is temporally stretched with a diffraction grating pair. This pulse is characterized using a chirp-scan type measurement, yielding a value of 75000 fs^2^ for the linear chirp, which is equivalent to 4 ps FWHM. Maximum pulse energies in this arm are 400 μJ. This pulse, which is used as the control field, is set at a time delay of about −1 ps with respect to the pump pulse to ensure that the 1.5 ps predissociation occurs during the flattest part of the control pulse. The instrument temporal response time, considered as the temporal cross-correlation of the pump and probe laser beams, was measured through multiphoton ionization of Xe, obtaining a value of ~ 400 fs.

### Velocity map imaging technique

All laser beams, with parallel polarizations, and controllable energies, focusing geometries and delays, are propagated collinearly and focused into the interaction region of the vacuum chamber with a 25 cm focal length lens. In the interaction region, they intercept a supersonic molecular beam of CH_3_I seeded in He produced by a 1 kHz piezoelectric valve. The generated ions in the interaction region are extracted perpendicularly to the plane defined by the laser propagation and the molecular beam, and are accelerated by means of an electrostatic lens system working in velocity map imaging configuration^42^, and mass separated in a 50 cm field-free region time-of-flight (TOF) mass spectrometer. The ion spheres are projected onto a time-gated microchannel plate (MCP) detector coupled to a phosphor screen. Finally, a Peltier-cooled 12 bit charge-coupled device (CCD) camera records the phosphorescence emitted by the phosphor screen as raw images that are later inverted using the Abel transform pBasex methodology^43^ to extract the desired information (translational energy and angular distribution).

### Data availability

The data that support the findings of this study are available from the corresponding author upon reasonable request.

## Electronic supplementary material

Supplementary Information
