# Supplementary material for: Strong laser field control of fragment spatial distributions from a photodissociation reaction
Source: Nat Commun. 2017 Nov 7;8:1345. doi: 10.1038/s41467-017-01139-6 (PMC5677097; doi:10.1038/s41467-017-01139-6)
Supplement: Supplementary file 1 — Supplementary Information [file 41467_2017_1139_MOESM1_ESM.pdf]

Supplementary Note 1 This material shows the quantitative values obtained in this work for the anisotropy parameters describing the angular distributions of the CH_3 fragments upon CH_3I dissociation in the presence of a NIR control field. β_2 and β_4 β_6 are shown for the weak field case (Supplementary Figure 1) and the strong field case (Supplementary Figure 2). It is useful to note that the asymptotic values obtained for these parameters in the strong field case, both for the predissociation channel and for the pump-dump channel, are significantly close to those defining the early stages (near zero delay) in the weak field case. As is described in detail in the Results section of the paper, this provides evidence that lifetime modification is the main mediator of angular distribution control in the present scheme.

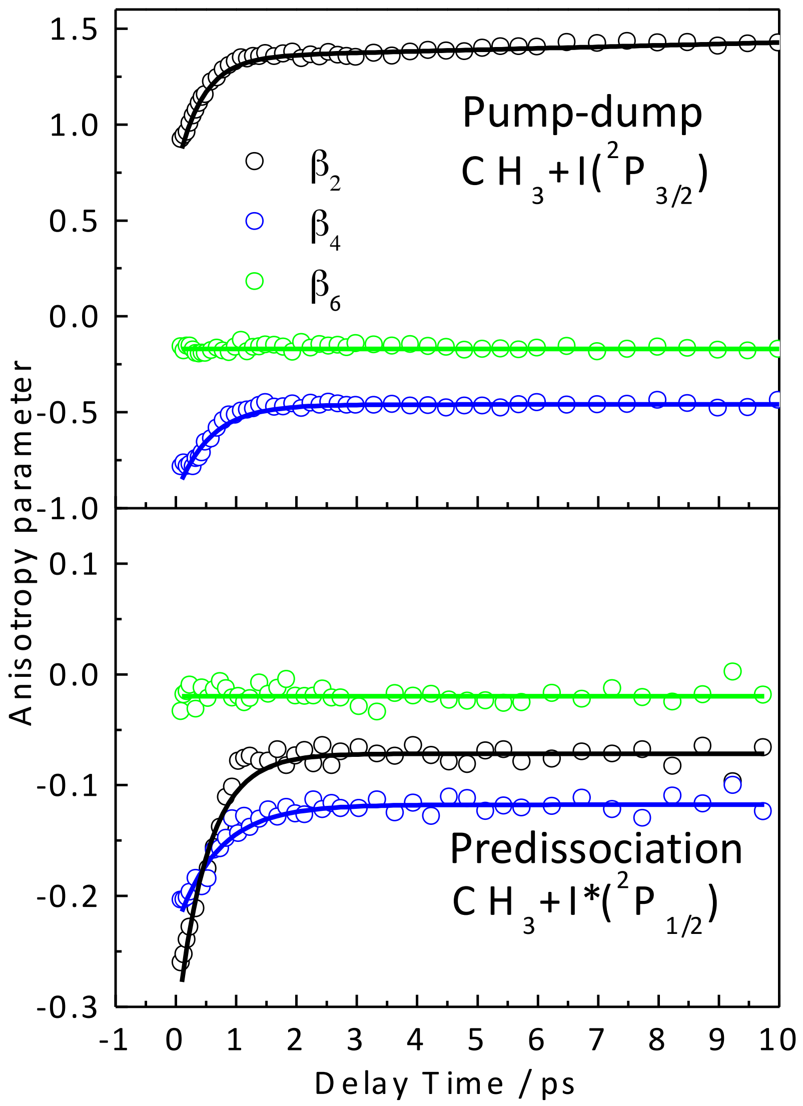

Supplementary Figure 1 Anisotropy parameters β_2, β_4 and β_6 measured as a function of time for the two channels (predissociation and pump-dump) in the presence of the low NIR control field (0.5 TW/cm^2.)

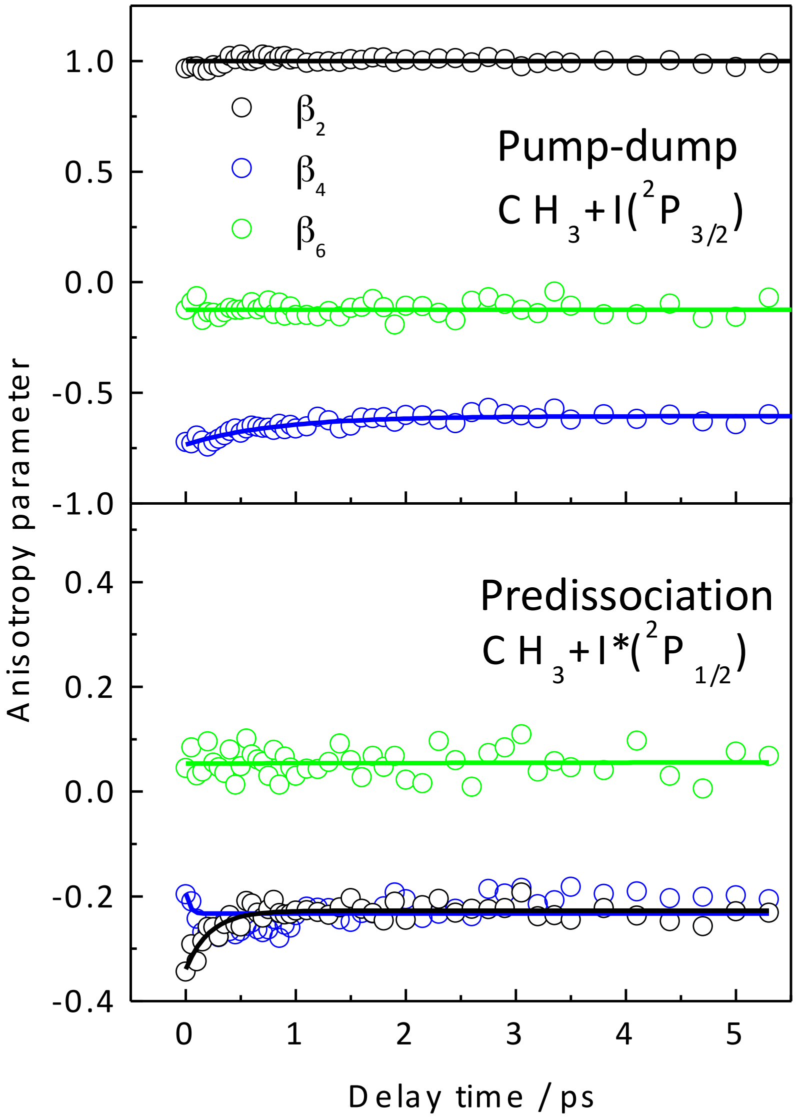

Supplementary Figure 2 Anisotropy parameters β_2, β_4 and β_6 measured measured as a function of time for the two channels (predissociation and pump-dump) in the presence of the high NIR control field (2.5 TW/cm^2.)
